# Supplementary material for: Protective Effect of Memantine on Bergmann Glia and Purkinje Cells Morphology in Optogenetic Model of Neurodegeneration in Mice
Source: Int J Mol Sci. 2021 Jul 22;22(15):7822. doi: 10.3390/ijms22157822 (PMC8346112; doi:10.3390/ijms22157822)
Supplement: Supplementary file 1 [file ijms-22-07822-s001.zip › ijms-1290174-supplementary.pdf]

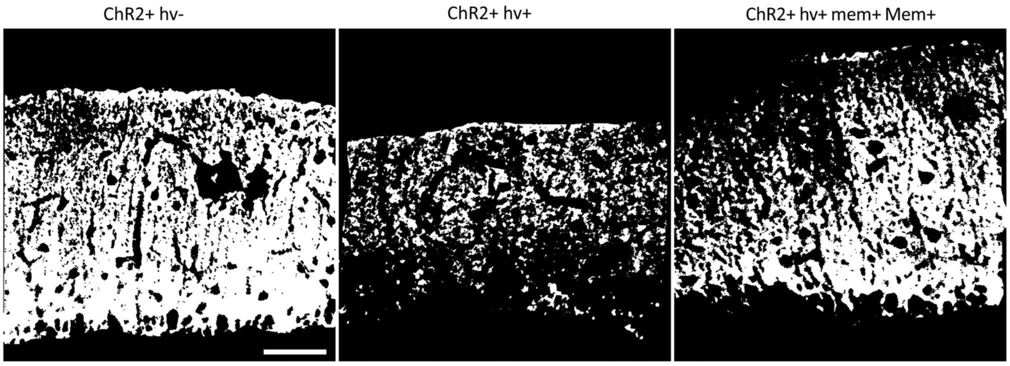

Figure S1. Contrast-enhanced microphotograph of the anti-EAAT1-labeled areas. Images contain digitally traced anti-EAAT1 fluorescence signals generated with Fiji software.
